# Incidence and Risk of Fatal Adverse Events in Cancer Patients Treated With Vascular Endothelial Growth Factor Receptor 2-Targeted Agents: A Meta-Analysis With Trial Sequential Analysis of Randomized Controlled Trials

**DOI:** 10.3389/fmed.2019.00176

**Published:** 2019-08-07

**Authors:** Bin Zhao, Hong Zhao, Jiaxin Zhao

**Affiliations:** ^1^The Second Affiliated Hospital and Yuying Children's Hospital, Wenzhou Medical University, Wenzhou, China; ^2^The Third Affiliated Hospital of Harbin Medical University, Harbin, China; ^3^The Fourth Affiliated Hospital of Harbin Medical University, Harbin, China; ^4^Heilongjiang Academy of Medical Sciences, Harbin, China

**Keywords:** vascular endothelial growth factor receptor 2, fatal adverse event, cancer, ramucirumab, apatinib

## Abstract

**Background/Aim:** Agents targeting vascular endothelial growth factor (VEGF) pathway have dramatically improved the outlook of cancer treatment. Meanwhile, it is well-known that they are associated with increases in the risk of fatal adverse events (FAEs). Vascular endothelial growth factor receptor 2 (VEGFR2)-targeted drugs have been approved for the treatment of several malignancies. However, little is known regarding the FAEs induced by VEGFR2-targeted agents across different tumor types and treatment regimens.

**Methods:** We searched PubMed and Embase database from January 1966 to April 2018 for randomized controlled trials (RCTs) to calculate the incidence and relative risks (RRs) of FAE.

**Results:** Seventeen RCTs involving 6,982 subjects with solid tumors were included in this study. The overall incidence of FAEs associated with VEGFR2-targeted agents was 1.7% (95% CI: 0.9–2.4%). Compared with controls, the administration of VEGFR2-targeted agents did not increase the risk of FAEs (RR, 1.29; 95% CI: 0.90–1.86). No significant association was found between FAE and VEGFR2-targeted agents in subgroup analyses based on tumor type, treatment strategy, clinical phase, masking method, median treatment duration, and approval status. Additionally, FAEs occurred in the major organ systems dispersedly. Trial sequential analysis revealed that our results are solid and further studies are unlikely to change this.

**Conclusions:** VEGFR2-targeted agents were not associated with an increased risk of FAEs.

## Introduction

Angiogenesis is a complicated process that plays a pivotal role in sustaining cancer microenvironment, tumor growth, and metastasis in many solid tumors (1). The vascular endothelial growth factor (VEGF) family, including several different VEGF isoforms and placenta growth factor, is one of the key mediators in this process (2). Accordingly, the VEGF pathway has been the leading target in cancer drug design and development. Currently, anti-VEGF agents, including small-molecule tyrosine kinase inhibitors (TKIs) such as sorafenib and sunitinib and monoclonal antibodies like bevacizumab and aflibercept, have been approved and widely used in cancer treatments. In addition, the VEGF pathway plays a key role in several physiological functions including tissue neovascularization, vascular, and cardiomyocyte homeostasis, and wound healing (3, 4). As a result, VEGF-targeted agents are often associated with a distinct profile of adverse events (AEs), and some AEs could be potentially life threatening. In fact, it is well-established that anti-VEGF agents are associated with increases in the relative risk (RR) of fatal adverse events (FAEs) compared with control (5–9).

VEGF in involved in the physiological function through binding to VEGF receptors (VEGFRs) on the cell surface. Furthermore, it has been revealed that the activation of VEGFR2 by VEGF is overwhelmingly regarded as the most critical driver of tumor angiogenesis (2). Since 2014, two VEGFR2-targets agents, namely ramucirumab and apatinib, have been approved by regulation authorities. These VEGFR2-targeted agents are still being investigated in various types of tumors and an increase in their application can be expected in the future. Although FAEs have occasionally been reported in subjects treated with ramucirumab, no significant, and definitive results have been established. Here, to examine the overall incidence and risk of FAEs associated with VEGFR2-targeted agents, we undertook the first meta-analysis among patients with solid tumors in randomized clinical trials (RCTs). Moreover, we applied trial sequential analysis (TSA) to investigate whether the currently available evidence was sufficient and conclusive.

## Materials and Methods

This study was reported according to the Preferred Reporting Items for Systematic Reviews and Meta-Analysis (PRISMA) statement (Supplementary Material) (10).

### Search Strategy

A systematic search of PubMed and Embase database from January 1966 to April 2018 was carried out without language restrictions. Considering a recent trial with VEGFR2-targeted agents had not been published, we also searched the abstracts from the European Society of Medical Oncology and American Society of Clinical Oncology Annual Meeting from January 2000 to April 2018. The keywords used were (1) vascular endothelial growth factor receptor 2, VEGFR2, kinase insert domain receptor, KDR, fetal liver kinase 1, Flk1; (2) ramucirumab, LY3009806, IMC-1121B, Cyramza; (3) apatinib, YN968D1. All investigators independently performed the initial search, carefully screened the titles and abstracts for relevance, and identified trials as excluded, included and uncertain. For those uncertain studies, the full texts were reviewed for confirmation of their eligibility. Any discrepancy was solved by discussion.

### Eligibility Criteria

**Figure 1:**
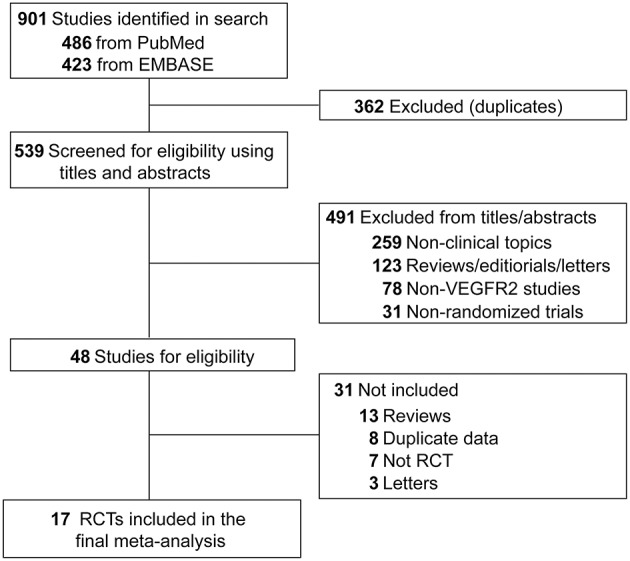
Flow-chart of eligible trials included in this study.

Both inclusion and exclusion criteria were pre-specified. To be eligible, studies had to meet the following criteria: (1) population: prospective randomized controlled trials (RCTs) involving adult patients with solid tumor; (2) intervention: random assignment of patients to VEGFR2-targeted agents or non-VEGF TKI control (chemotherapy or placebo) alone or in combination with other treatment; (3) outcomes: available information on sample size and FAEs. Other studies on this topic, including phase 1 trials, review articles, pre-clinical papers, early versions of data later published, and editorials were not included (Figure 1). When multiple publications of the same study occurred, only the most recent and/or most complete reporting study was included.

### Data Extraction and Quality Assessment

Eligible studies were collected and full texts were examined for the trial design and reporting of FAEs. The following items were extracted: first author's name, year of publication, clinical phase, masking method, tumor type, number of patients enrolled, number of patients for safety analysis, therapy strategy, median treatment duration, median follow-up, number of FAEs, and approval status (Table 1). All data were extracted independently by all the reviewers, and any discrepancies were settled by discussion and consensus.

**Table 1:** Characteristics of trials included in this study.

**Refrences**	**Trial phase**	**Mask method**	**Tumor type**	**No. of patients enrolled**	**No. of patients (safety)**	**Treatment**	**Median treatment duration (weeks)**	**Median follow-up (months)**	**FAE**	**Approval status**	**Jadad score**
Petrylak et al. (11)	3	Double blind	UC	263	258	RAM 10 mg/kg + docetaxel 75 mg/m^2^ on day 1 of 21-day cycle	12.1	5.0	8	No	4
				267	265	Placebo + docetaxel 75 mg/m^2^ on day 1 of 21-day cycle	9.9		5		
Fuchs et al. (12)	3	Double blind	GC/GJC	238	236	RAM 8 mg/kg once of 14-day cycle	8.0	<28.0	5	Yes	5
				117	115	Placebo once of 14-day cycle	6.0		2		
Garon et al. (13)	3	Double blind	NSCLC	628	627	RAM 10 mg/kg + docetaxel 75 mg/m^2^ on day 1 of 21-day cycle	15.0	9.5	15	Yes	5
				625	618	Placebo + docetaxel 75 mg/m^2^ on day 1 of 21-day cycle	12.0	8.8	9		
Wilke et al. (14)	3	Double blind	GC/GJC	330	327	RAM 8 mg/kg on day 1,15+ paclitaxel 80 mg/m^2^ on day 1,8,15 of 28-day cycle	18.0	7.9	6	Yes	5
				335	329	Placebo + paclitaxel 80 mg/m^2^ on day 1,8,15 of 28-day cycle	12.0		5		
Mackey et al. (15)	3	Double blind	BC	759	752	RAM 10 mg/kg + docetaxel 75 mg/m^2^ on day 1 of 21-day cycle	28.0	18.6	0	No	4
				385	382	Placebo + docetaxel 75 mg/m^2^ on day 1 of 21-day cycle	27.0		0		
Zhu et al. (16)	3	Double blind	HCC	283	277	RAM 8 mg/kg once of 14-day cycle	12.0	8.3	7	No	4
				282	276	Placebo 8 mg/kg once of 14-day cycle	8.0	7.0	4		
Tabernero et al. (17)	3	Double blind	CRC	536	529	RAM 8 mg/kg + FOLFIRI once of 14-day cycle	19.0	21.7	13	No	5
				536	528	Placebo 8 mg/kg + FOLFIRI once of 14-day cycle	18.0		10		
Doebele et al. (18)	2	Open label	NSCLC	69	67	RAM 10 mg/kg, pemetrexed 500 mg/m^2^, carboplatin or cisplatin 75 mg/m^2^ on Day 1 of 21-day cycle	15.0	>24.0	2	No	3
				71	69	Placebo, pemetrexed 500 mg/m^2^, carboplatin or cisplatin 75 mg/m^2^ on Day 1 of 21-day cycle	12.0		5		
Petrylak et al. (19)	2	Open label	UC	46	46	RAM 10 mg/kg + docetaxel 75 mg/m^2^ on day 1 of 21-day cycle	9.1	<42.0	0	No	2
				45	45	Placebo + docetaxel 75 mg/m^2^ on day 1 of 21-day cycle	14.3		0		
Moore et al. (20)	2	Open label	CRC	52	52	RAM 8 mg/kg + mFOLFOX-6 on day 1 of 14-day cycle	16.0	<24.0	2	No	3
				54	49	Placebo + mFOLFOX-6 on day 1 of 14-day cycle	15.3		0		
Hussain et al. (21)	2	Open label	PC	66	66	RAM 6 mg/kg on day 1,8,15 of 21-day cycle	19.0	<32.0	2	No	2
				66	66	Cixutumumab 6 mg/kg on day 1,8,15 of 21-day cycle	15.0		1		
Vahdat et al. (22)	2	Open label	BC	52	52	RAM 10 mg/kg on day 1, 8 + capecitabine 2000 mg/m^2^ on day 1–14 of 21-day cycle	14.0	<24.0	1	No	3
				49	49	Placebo + capecitabine 2000 mg/m^2^ on day 1–14 of 21-day cycle	6.0		0		
Yardley et al. (23)	2	Open label	BC	71	69	RAM 10 mg/kg on day 1+ eribulin 1.4 mg/m^2^ on day 1,8 of 21-day cycle	12.0	NR	2	No	3
				70	65	Placebo on day 1+ eribulin 1.4 mg/m^2^ on day 1,8 of 21-day cycle	27.0		1		
Yoh et al. (24)	2	Double blind	NSCLC	76	76	RAM 10 mg/kg + docetaxel 60 mg/m^2^ on day 1 of 21-day cycle	13.0	<30.0	1	No	3
				81	81	Placebo + docetaxel 60 mg/m^2^ on day 1 of 21-day cycle	12.6		1		
Yoon et al. (25)	2	Double blind	GC/EC	84	82	RAM 8 mg/kg + mFOLFOX-6 on day 1 of 14-day cycle	21.0	<30.0	0	No	4
				84	80	Placebo + mFOLFOX-6 on day 1 of 14-day cycle	25.0		3		
Li et al. (26)	2	Double Blind	GC	47	47	Apatinib 850 mg once daily of 28-day cycle	NR	<14.0	0	No	3
				48	48	Placebo once daily of 28-day cycle	NR		0		
Li et al. (27)	3	Double blind	GC/GJC	176	176	Apatinib 850 mg once daily of 28-day cycle	11.6	<27.0	0	Yes	4
				91	91	Placebo once daily of 28-day cycle	7.6		0		

*BC, breast cancer; CRC, colorectal cancer; EC, esophageal cancer; GC, gastric cancer; GJC, gastro-esophageal junction cancer; HCC, hepatocellular carcinoma; NSCLC, non-small-cell lung cancer; PC, prostate cancer; UC, urothelial cancer; RAM, ramucirumab; FAE, fatal adverse event; FOLFIRI, irinotecan 180 mg/m^2^, levo-leucovorin 200 mg/m^2^ followed by fluorouracil 400 mg/m^2^; mFOLFOX-6, Oxaliplatin 85 mg/m^2^ and leucovorin 400 mg/m^2^ followed by 5-FU 400 mg/m^2^; NR, not reported*.

The quality of eligible trials was evaluated by the seven-item Jadad scale including randomization, double blinding, and withdrawals as previously described (28).

### Trial Sequential Analysis

In any single trial, interim analyses can increase the risk of Type I error (false-positive results). To avoid it, monitoring boundaries has been used to examine whether a study could be stopped early because the *p* value was small enough to show the anticipated effect or for futility. Similarly, meta-analysis may also result in type I errors because of sparse data and/or repetitive examining (29). Because no reason exists for the standards for a meta-analysis to be less rigorous than those in a single trial, trial sequential monitoring boundaries were introduced (29, 30). This allows to evaluate whether the results from the meta-analysis are reliable and conclusive. When the cumulative z curve crosses the trial sequential monitoring boundary or enters the futility area, a sufficient level of evidence for the anticipated intervention effect may have been reached and no further trials are needed. If the z curve crosses none of the boundaries and the required information size has not been reached, there is insufficient evidence to reach a conclusion. Here, we estimated the required information size using α = 0.05 (two-sided), β = 0.20 (power of 80%). Trial sequential analysis was conducted by TSA version 0.9.5.9 Beta (http://www.ctu.dk/tsa).

### Statistical Analysis

The primary aim is to examine the overall incidence, relative risk (RR) and corresponding 95% confidence intervals (CIs) of FAEs in cancer patients treated by VEGFR2-targeted agents. To calculate the incidence, the number of patients receiving VEGFR2-targeted agents and the number of FAEs were extracted from eligible studies. For the calculation of RR, patients treated with VEGFR2-targeted agents were compared with those assigned to control arm in the same trial. When trials reported no FAE in one arm, a classic half-integer continuity correction was used to calculate RR.

Statistical heterogeneity across trials was evaluated by Cochrane's Q statistic. The *I*^2^ statistic was calculated to assess the extent of inconsistency contributable to the heterogeneity across different studies (31). The assumption of homogeneity was considered invalid for *I*^2^> 25% or *p* < 0.05. Summary RRs and incidences were calculated using fixed-effects model or random-effects model depending on the heterogeneity of included trials. To check the impact of various clinicopathological variables on FAE, we further conducted *post hoc* subgroup analysis based on various VEGFR2-targeted agents, underlying malignancy, treatment strategy, clinical phase, masking method, median treatment duration, and approval status.

Potential publication bias was assessed by visual inspection of a funnel plot, and also evaluated using the tests of Egger et al. (32) and Begg et al. (33). Two-sided *p* < 0.05 were considered statistically significant. All analyses were conducted by MedCalc 13.0 (MedCalc Software, Belgium) and Stata 12.0 (StataCorp, USA).

## Result

### Search Results

A total of 901 potentially relevant articles were identified from the initial search, including 486 studies from PubMed and 423 trials from Embase database. Three hundred and sixty-two articles were excluded because of duplications. After careful screening of the titles and abstracts, 491 studies were removed. After further reviewing the complete texts of the remaining 48 potentially eligible articles, 17 RCTs were enrolled for the final analysis (Figure 1). Ramucirumab was studied in 15 trials, apatinib was examined in two RCTs (Table 1).

### Study Quality

Randomized treatment allocation sequences were generated in all trials. Eight studies were phase three RCTs, while the remaining nine studies were phase 2 trials. Eleven trials were double-blinded, six studies were open labeled. Sample size and FAEs were reported in all the 17 included trials. Additionally, the follow-up time was adequate for every RCT. We further graded the quality of each trial by the 7-item Jadad score which can provide a score ranging from 0 to 5 for every RCT. All RCTs included in this study had a score of 2–5 indicating moderate or good quality. The association of VEGFR2-targeted agents with FAE did not show significant difference with Jadad score (score ≤ 3 vs. score > 3; *P* = 0.51).

### Patients

A total of 6,982 patients were enrolled in the eligible 17 RCTs. All the subjects in these trials were over 18 years old, had adequate renal, hepatic, cardiac, and hematologic function. Safety population consisted of 6,895 subjects (VEGFR2-targeted agents, 3,739; control, 3,156). Underlying malignancies included gastric cancer/gastro-esophageal junction cancer/esophageal cancer (five trials) (12, 14, 25–27), breast cancer (three trials) (15, 22, 23), Non-small-cell lung cancer (three trials) (13, 18, 24), colorectal cancer (two trials) (17, 20), urothelial cancer (two trials) (11, 19), hepatocellular cancer (one trial) (16), and prostate cancer (one trial) (21).

### Incidence of FAEs

Totally, there were 110 FAEs (VEGFR2-targeted agents, 64; control, 46) among 6,895 patients. Using a random-effects model (heterogeneity test: *Q* = 53.17; *P* < 0.001; *I*^2^ = 69.9%), the summary incidence of FAEs in patients receiving VEGFR2-targeted agents was 1.7% (95% CI: 0.9–2.4%). We further examined the possible reasons for this heterogeneity. As shown in Table 2, the incidences of FAEs differed significantly by tumor type (*p* = 0.046) and masking method (*p* = 0.017), indicating the contributions of these factors to the incidence of FAEs were varied in patients treated with VEGFR2-targeted agents.

**Table 2:** Incidence and relative risk (RR) of FAE associated with VEGFR2-targeted agents according to underlying malignancy, treatment strategy, clinical phase, masking method, median treatment duration, and approval status.

	**No. of Trials**	**No. of FAEs/No. of patients**		**Incidence of FAE, % (95% CI)**		**RR (95% CI)**
		**VEGFR2**	**Control**	**VEGFR2**	**Control**	
**Underlying malignancy**						
GC/GJC/EC	5	11/868	10/663	1.3 (0.6–2.0)	1.5 (0.7–2.4)	0.86 (0.38–1.94)
Non-small-cell lung cancer	3	18/770	15/768	2.3 (1.5–3.0)	2.0 (1.1–2.9)	1.20 (0.61–2.37)
Breast cancer	3	3/873	1/496	0.3 (0.1–0.6)	0.2 (0.0–0.4)	2.13 (0.44–10.38)
Colorectal cancer	2	15/581	10/577	2.6 (1.4–4.9)	1.7 (0.8–2.6)	1.47 (0.67–3.18)
Urothelial cancer	2	8/304	5/310	2.6 (0.4–5.7)	1.6 (0.0–3.3)	1.58 (0.55–4.56)
Hepatocellular cancer	1	7/277	4/276	2.5 (0.0–1.0)	1.5 (0.0–0.0)	1.97 (0.68–8.73)
Prostate cancer	1	2/66	1/66	3.0 (0.9–5.2)	1.5 (0.0–3.1)	1.93 (0.77–12.05)
**Treatment strategy**						
Combination therapy	12	50/2,937	39/2,560	1.7 (0.9–2.5)	1.5 (0.7–2.2)	1.25 (0.86–1.91)
Monotherapy	5	14/802	7/596	1.7 (0.8–2.5)	1.2 (0.6–1.8)	1.48 (0.64–3.46)
**Clinical phase**						
Phase II	9	10/557	11/552	1.8 (0.7–2.9)	2.0 (0.9–3.0)	0.92 (0.43–1.97)
Phase III	8	54/3,182	35/2,604	1.7 (0.9–2.5)	1.3 (0.6–2.0)	1.43 (0.94–2.17)
**Masking method**						
Double blind	11	55/3,387	39/2,813	1.6 (0.9–2.1)	1.4 (0.8–1.9)	1.31 (0.88–1.95)
Open label	6	9/352	7/343	2.6 (1.5–3.7)	2.0 (1.1–2.9)	1.22 (0.50–3.00)
**Median treatment duration**						
<15 weeks	8	24/1,190	13/987	2.0 (1.1–3.0)	1.3 (0.7–1.9)	1.54 (0.82–2.92)
≥15 weeks	8	40/2,502	33/2,121	1.6 (1.1–2.2)	1.6 (0.9–2.3)	1.19 (0.76–1.85)
**Approval status**						
Approved	4	26/1,366	16/1,153	1.9 (1.1–2.7)	1.4 (0.8–2.0)	1.41 (0.77–2.59)
Not approved	13	38/2,373	30/2,003	1.6 (1.2–2.0)	1.5 (0.9–2.1)	1.23 (0.78–1.94)
**Overall**	**17**	**64/3,739**	**46/3,156**	**1.7 (0.9–2.4)**	**1.5 (0.8–2.1)**	**1.29 (0.90–1.86)**

*CI, confidence interval; EC, esophageal cancer; GC, gastric cancer; GJC, gastro-esophageal junction cancer; FAE, fatal adverse event. The bold values indicate the pooled numbers calucultad by data extracted from all the included trials*.

### RR of FAEs

**Figure 2:**
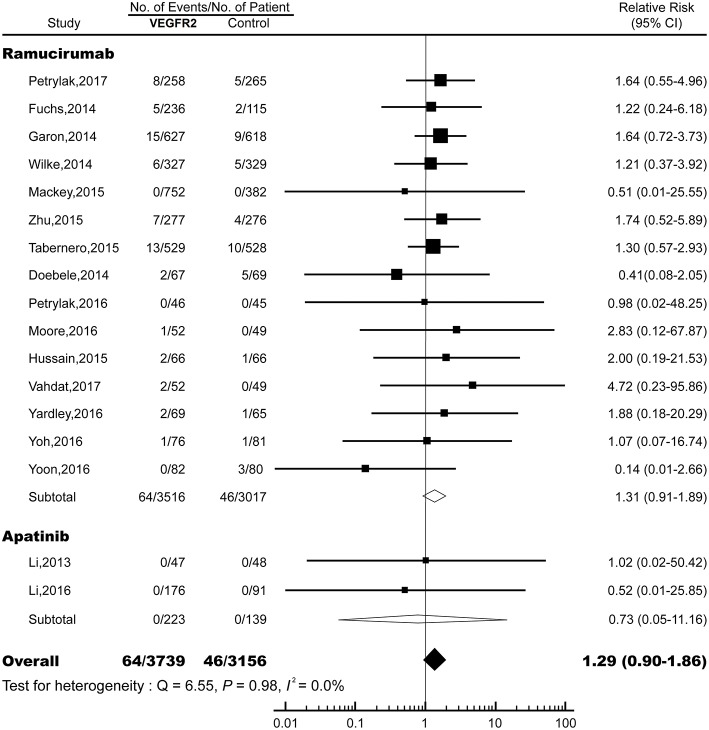
Relative risk (RR) of fatal adverse events (FAEs) associated with VEGFR2-targeted agents vs. control. Overall risk of FAEs was calculated by fixed-effects models.

The overall RR of FAE induced by VEGFR2-targeted agents from 17 RCTs was 1.29 (95% CI: 0.90–1.86; *P* > 0.05; Figure 2), indicating the risk of FAE in subjects treated with VEGFR2-targeted was not statistically different from those in the control arms. No significant heterogeneity was identified (*Q* = 6.55; *I*^2^ = 0.0%; *P* = 0.98). We also explored the relationship between the RR of FAEs with VEGFR2-targeted agents stratified by tumor type, treatment strategy, clinical phase, masking method, median treatment duration, and approval status (Table 2). No significant association was found in all these subgroup analyses.

**Figure 3:**
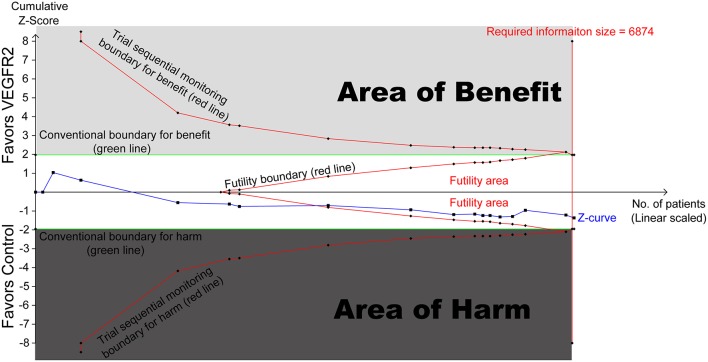
Trial sequential analysis (TSA) of 17 RCTs comparing VEGFR2-targeted agents with control (scaled trial distance). A diversity-adjusted required information size of 6,874 patients was calculated using α = 0.05 (two-sided) and β = 0.20 (power of 80%), an anticipated relative risk reduction of 20% in the control arm. TSA of 17 trials (black filled squares) demonstrating that the cumulative *z* curve crossed the futility boundary and required information size line, establishing conclusive and sufficient evidence and suggesting no further trials are needed. X axis, number of patients randomized; Y axis, cumulative z score; horizontal green dotted lines, conventional boundaries (z score, ±1.96; two-sided *p* = 0.05); Sloping red lines with black filled circles, trial sequential monitoring boundaries; blue line with black filled squares, *z* curve; vertical red line, required information size; upper light-gray rectangle, area of benefit; lower dark-gray rectangle, area of harm; middle white rectangle, futility area.

To evaluate the reliability of this meta-analysis, TSA was conducted. As shown in Figure 3, the cumulative z curve first crossed the futility boundary and entered the futility area, then crossed the required information size line, which established sufficient and conclusive evidence. Thus, further trials were not needed and were unlikely to change our conclusions.

### Specific FAEs Caused by VEGFR2-Targeted Agents

Out of the 64 FAEs associated with VEGFR2-targeted agents, 16 (25.0%) had unknown or unspecified causes, while the reason for the remaining 48 mortalities were reported. FAEs dispersedly occurred in the major organ systems. The most frequently occurred FAEs were sepsis and infection, representing a total of 12 deaths or 25.0% of all specific FAEs. Other common recorded FAEs were hemorrhage (*n* = 8, 16.7%), respiratory events (*n* = 7, 14.6%), gastrointestinal events (*n* = 7, 14.6%), cardiovascular events (*n* = 7, 14.6%), hepatic events (*n* = 4, 8.3%), and renal events (*n* = 2, 4.2%).

### Publication Bias

There was no evidence of publication bias by inspection of the funnel plot and formal statistical tests (Begg's test, *P* = 0.76; Egger's test, *P* = 0.82). Visual inspection of Begg's funnel plot also did not identify substantial asymmetry.

## Discussion

To our knowledge, this is the first meta-analysis focused especially on the incidence and risk of FAEs in patients treated with VEGFR2-targeted agents. Based on 17 RCTs, our result revealed that the incidence of FAEs in cancer patients treated with VEGFR2-targeted agents was 1.7% compared with 1.5% in subjects from control/placebo arms. Additional analysis showed there was no association between VEGFR2-targeted treatment and risk of FAEs in patients with solid tumors. The process by which subject doctors in studies went about determining whether a death was the result of VEGFR2-targeted agent or just a subjective process and certainly a potential source of bias should not play a key role since analysis on those double-blinded and high-quality RCTs yielded similar results. In fact, we could not observe any significant differences in the subgroup analysis conducted in this study. Moreover, TSA confirmed that our results are solid and reliable, and further studies are not needed.

Drugs targeting VEGF pathway have dramatically improved the outlook of cancer treatment in the past several decades. Meanwhile, since these agents inhibit the growth of blood vessels, it is well-known that they are associated with increases in the risk of treatment related mortality compared with control (5–8). For example, in advanced non-small cell lung cancer, the administration of anti-VEGFR agents could significantly increase the risk of FAEs (34). It should be noted that in this study, the first generation agents targeting VEGFR such as sorafenib, sunitinib, and axitinib were included for analysis. While in gastric cancer, because only bevacizumab and ramucirumab were investigated, no association was found between molecular targeted agents and FAEs (35). Here, we summarized the incidences and risks of FAE in patients treated with several widely used agents. As shown in Table 3, the incidence and relative risk varied among different anti-angiogenic agents. A previous study showed that the addition of bevacizumab, a monoclonal antibody included in the World Health Organization's list of essential medicines, was associated with an increased risk of FAEs (RR, 1.33; 95% CI: 1.02–1.73) compared with control (6). Sorafenib, a small molecule that antagonizes the intracellular domain of the VEGFR and blocks the downstream signaling, could also significantly increase the risk of FAEs (RR, 1.82; 95% CI: 1.05–3.14) (7). In contrast, our results revealed that there was no difference between patients treated VEGFR2-targeted agents and those in the control arms in terms of FAE risk. This might suggest that VEGFR2-targeted agents were safer than other anti-angiogenic agents. The mechanisms underlying these discrepancies remain unknown. However, it cannot be ruled out that the differences between VEGFR2-targeted agents and other anti-angiogenics may be due to patient population, tumor type, mechanisms of action, dosage, and treatment duration. Interestingly, it was reported that the risk of developing proteinuria, hypertension, gastrointestinal perforation, infusion related reactions, reversible posterior leukoencephalopathy syndrome, wound healing delay, and all-grade bleeding in patients treated with ramucirumab were consistent with those in the angiogenesis inhibitor class (36). However, no evidence for increased risk of arterial thromboembolic events, venous thromboembolic events, or high-grade bleeding was discovered (36), suggesting that ramucirumab may be distinct among anti-angiogenic agents in relation to thromboembolism and bleeding. Considering the most common causes of FAEs in patients treated with anti-angiogenics were hemorrhage and cardiac events (5–8), it may partly explain the relatively low risk of treatment related mortalities.

**Table 3:** Overall incidence and relative risk of fatal adverse events in patients treated with anti-angiogenic agents.

**Agent**	**Target**	**Incidence (%, 95% CI)**	**Relative risk (95% CI)**	**References**
Aflibercept	VEGF	5.1 (3.8–6.8)	1.81(1.20–2.72)	(5)
Bevacizumab	VEGF	2.9 (2.0–4.2)	1.33(1.02–1.73)	(6)
Sorafenib	VEGFR/PDGFR	1.3 (0.8–2.2)	1.82(1.05–3.14)	(7)
Sunitinib	VEGFR/PDGFR	1.2 (0.7–1.8)	2.34(1.34–4.09)	(9)
Ramucirumab	VEGFR2	1.9 (1.1–3.0)	1.31(0.91–1.89)	Current study

*CI, confidence interval; PDGFR, placenta-derived growth factor receptor; VEGF, vascular endothelial growth factor; VEGFR, vascular endothelial growth factor receptor*.

Our study has important clinical implications. It is reported that mortality associated with adverse drug reactions accounts for ~5% of all hospital fatalities (37, 38). Accordingly, the benefit/risk evaluation should play an essential role in the decision-making process during cancer treatments selection. For anti-angiogenic agents, patients should recognize the increased risk of treatment related mortality before consenting to these kinds of targeted cancer therapy. Our study could be important in considering the benefit/risk trade-off by providing the overall incidence and relative risk of FAEs in patients treated with VEGFR2-targeted agents.

Our meta-analysis has several strengths. We performed a comprehensive review, utilized the most up-to-date published data. All the included original studies are phase II or phase III RCTs, which minimized selection bias. Moreover, with the accumulating evidence and enlarged sample sizes (i.e., the study population was similar to the general population), this study enhanced the statistical power with more reliable and precise clinical outcome estimates. Additionally, to increase the robustness of our study, we conducted several subgroup analyses stratified by tumor type, VEGFR2-targeted agents, treatment strategy, clinical phase, masking method, median treatment duration, and approval status. TSA was also applied to evaluate the impact of repetitive testing and random errors.

This study also has some limitations. First, our study is based on data from clinical trials rather than individual patients. This may include some confounding factors such as previous therapies received, patients' comorbidities, and concomitant medications. Second, it is important to emphasize that subjects who are eligible for RCTs show normal functions of major organs, which could result in underestimating the risk of bleeding and cardio-toxicity in real-world clinical practice. Third, some trials included were open labeled RCTs. Even for those double-blinded trials, skillful clinicians might identify AEs induced by VEGFR2-targeted agents. This might lead to potential bias. Forth, the incidences of FAEs among the included studies had significant heterogeneity. Here we adjusted this heterogeneity by performing a random-effects model to calculate the overall incidence. Even so, it might underestimate the real event rate since trials without any death could receive disproportional weight in calculation.

In summary, the administration of VEGFR2-targeted agents does not increase the risk of FAEs. Accordingly, the benefit/risk should be properly weight by both practitioners and patients in drug selection.

## Data Availability

The raw data supporting the conclusions of this manuscript will be made available by the authors, without undue reservation, to any qualified researcher.

## Author Contributions

BZ study design, meta-analysis, wrote, and reviewed the manuscript. HZ and JZ database management and search strategies, wrote, and reviewed the manuscript.

### Conflict of Interest Statement

The authors declare that the research was conducted in the absence of any commercial or financial relationships that could be construed as a potential conflict of interest.
